# Language and Cognition

**DOI:** 10.3389/fnbeh.2014.00436

**Published:** 2014-12-16

**Authors:** Leonid Perlovsky, Kuniyoshi L. Sakai

**Affiliations:** ^1^Department of Electrical Engineering, School of Engineering and Applied Sciences, Harvard UniversityCambridge, MA, USA; ^2^Department of Basic Science, Graduate School of Arts and Sciences, The University of TokyoTokyo, Japan

**Keywords:** language, cognition, brain, functional imaging, emotion

Interaction between language and cognition remains an unsolved scientific problem. What are the differences in neural mechanisms of language and cognition? Why do children acquire language by the age of six, while taking a lifetime to acquire cognition? What is the role of language and cognition in thinking? Is abstract cognition possible without language? Is language just a communication device, or is it fundamental in developing thoughts? Why are there no animals with human thinking but without human language? Combinations even among 100 words and 100 objects (multiple words can represent multiple objects) exceed the number of all the particles in the Universe, and it seems that no amount of experience would suffice to learn these associations. How does human brain overcome this difficulty?

Since the nineteenth century we know about involvement of Broca's and Wernicke's areas in language. What new knowledge about the brain regions responsible for language and cognition has been found with fMRI and other brain imaging methods? Every year we know more about their anatomical and functional/effective connectivity. What can be inferred about their interactions and functions in language and cognition? Why does the human brain show hemispheric (i.e., left or right) dominance for some specific linguistic and cognitive processes? Is linguistic and cognitive comprehension processed in the same or different regions? Do the syntactic processes affect the structure of our conceptual world?

Such issues regarding brain functions and mind have been increasingly drawing attention from various fields in recent years, and investigations that go beyond the boundaries of previous fields of study are becoming necessary. The need for study spanning the brain and the mind has given birth to a new discipline, such as cognitive neuroscience, neurolinguistics, biolinguistics, etc. We assume that mind is a part of brain function, and we tentatively define the mind as a combination of three main cognitive factors: perception, memory, and consciousness. Language is created by mind, yet, once uttered, words return to the mind, where they are understood. The cycle from the mind to the language and then from the language to the mind, is *recursive*, in that the language produced by the mind comes back to the mind once again. This recursiveness is important when considering the relationship between language and mind.

When viewed language and mind as a whole system, it is evident that the functions of language are part of the brain system at the same time as being involved in the workings of the mind. Moreover, information is exchanged between language and each of perception, memory, and consciousness in both directions. Namely, language is involved in both reciprocal and recursive information exchange with each element of the mind. Since language is tightly linked to the mind, it would be more natural to assume that language is a part of the mind than to think it is an entity which exits outside the mind. The study of language is, in essence, to understand a part of the “human” mind. The more we study the language used by humans, the more we will understand the structure of the mind.

Chomsky has suggested that language is separable from cognition (Berwick et al., 2013), and this notion has been well supported by functional imaging experiments in neuroscience (Sakai, 2005). On the opposite, cognitive and construction linguistics emphasized a single mechanism of both. Neither has led to a computational theory so far, but language is learned early in life with only limited cognitive understanding of the world (Perlovsky, 2009). Evolutionary linguistics has emphasized evolution leading to a mechanism of language acquisition, yet proposed approaches also lead to incomputable complexity. Papers in this volume report new knowledge on interacting language and cognition, still there remains more questions than answers.

In animals, emotional and conceptual contents of voice sounds are fused. Evolution of human language has demanded splitting of emotional and conceptual contents, as well as of their mechanisms, although language prosody still carries emotional content. Is it a dying-off remnant, or is it fundamental for interaction between language and cognition? If language and cognitive mechanisms differ, unifying these two contents requires motivation, hence emotions. What are these emotions? Can they be measured? If tonal languages use pitch contours for semantic contents, are there differences in language-cognition interaction among tonal and atonal languages? Are emotional differences among cultures exclusively cultural, or also depend on languages?

This volume introduces a broad range of research addressing these topics, including three opinion articles, one hypothesis and theory article, eight original research articles, and a pair of an opinion article and a general commentary article. Their summaries are as follows.

First, Perlovsky (2013) introduces joint acquisition, dual hierarchy, and emotional prosody of language and cognition, such that emotional prosody may perform a fundamental function in connecting sounds and meanings of words. Vicario (2013) discusses about FOXP2 gene and language development, which might inform us about the origin of language. Perry and Lupyan (2013) explain that language and thought are different but strongly interacting abilities, based on the online manipulation of linguistic activity.

Next, Ohta et al. (2013) propose computational principles of syntax in the regions specialized for language, thereby integrating theoretical linguistics and functional neuroimaging. Nagels et al. (2013b) present an fMRI study on the neural substrates of figurative language during natural speech perception. De La Cruz et al. (2013) show that finger counting helps cognitive robots to learn words. Straube et al. (2013) suggest that abstract information conveyed by speech and gesture may be processed independent of modality. Tilles and Fontanari (2013) examine reinforcement and inference in cross-situational word learning. Nagels et al. (2013a) indicate the role of semantic abstractness and perceptual category in processing speech accompanied by gestures. Zhong et al. (2013) study a self-organizing pre-symbolic neural model representing sensorimotor information. Shuai and Gong (2013) analyze temporal relationships between top-down and bottom-up processing in lexical tone perception. Vicario and Rumiati (2013) demonstrate how notions of left and right affect processing of trading verbs.

We end the volume with a highly-popular discussion on the role of open access publications in linguistics, contributed by Haspelmath (2013) and Bragazzi (2013).

## Conflict of interest statement

The authors declare that the research was conducted in the absence of any commercial or financial relationships that could be construed as a potential conflict of interest.
